# Increasing Resident Wellness Through a Novel Retreat Curriculum

**DOI:** 10.7759/cureus.1524

**Published:** 2017-07-28

**Authors:** Angela Cornelius, Brian G Cornelius, Mary Ann Edens

**Affiliations:** 1 Department of Emergency Medicine, Louisiana State University Health Science Center Shreveport; 2 Department of Anesthesia, Crna, University Health Shreveport

**Keywords:** wellness, resident wellness, residency retreat, wellness curriculum, residency team building

## Abstract

Background

Because of their arduous schedules, residents are susceptible to burnout, fatigue, and depression. In 2015, the Accreditation Council for Graduate Medical Education (ACGME) launched a campaign to foster physician wellness, in response to the suicides of three residents during the previous year. The campaign calls for strategies to developing resiliency, identify problems, and promote well-being. One of the suggested methods to promote well-being was a residency retreat.

Objective

To implement a novel retreat curriculum that emphasizes team building between residents and faculty, with which residents expressed high satisfaction.

Methods

We created an "Amazing Race" style retreat involving five activity stations set up in a neighborhood park in which 25 of our 34 residents participated. These stations implemented team building, faculty-resident bonding and resident-resident bonding.

An anonymous survey was administered to the 25 participating emergency medicine (EM) residents after the retreat, of whom 21 returned the survey. The survey consisted of questions to assess the resident’s perception of the team building activities, their satisfaction with each of the five activity stations and overall retreat satisfaction.

Results

Of the 25 residents who participated in the retreat, 21 (84%) returned the post-retreat survey (one participant returned a survey leaving the ranking questions incomplete). This low-cost event received high satisfaction ratings in regard to team-building, resident bonding, and faculty-resident bonding.

Conclusions

This novel retreat proved to be a low-cost and easily implemented activity with which the residents expressed high levels of satisfaction.

## Introduction

United States emergency medicine (EM) residencies are a three-to-four-year undertaking, during which residents are taught clinical emergency medicine, taking the residents through almost all of the specialties that medicine has to offer. EM residents are likely to spend much more time with residents from other services than with residents from their department, making them feel disconnected from their direct colleagues and departmental faculty. In addition, many residents move across the country for residencies, and those that do not find the increased amount of time spent at work separates them from their established home social networks. Isolation from their usual social networks has been theorized to be linked to depression and suicide in physicians [1].

In 2015, the Accreditation Council for Graduate Medical Education (ACGME) launched a campaign to foster resident wellness, in response to the suicides of three residents in a short period during 2014. The campaign centers on developing resiliency, identifying indications of problems, promoting well-being, and learning from other tragedies to help grieving communities [2]. Also in 2015, a national panel of United States multispecialty residents and fellows recommended an increase in resident wellness activities, naming resident retreats specifically [3].

The Merriam-Webster dictionary defines a retreat as “a period of group withdrawal for prayer, meditation, study, or instruction under a director” [4]. A retreat provides bonding time with their direct colleagues, which fosters physician wellness as well as program wellness, especially when faculty members are involved. This outside bonding time helps residents build better working relationships with their direct peers, which lowers burnout rates [5].

A literature review revealed that 42 PubMed articles regarding resident wellness and/or burnout were published in the last two years. These articles were from multiple different medical specialties in many countries. Many were addressing wellness programs but no literature was found which addressed using a retreat as a wellness tool, such as we have done in this article. Only one abstract was found regarding an EM residency retreat being used for wellness [6].

Annually, since our residency program’s inception in 2004, we hold a five-hour retreat that is organized by the incoming chief residents as a part of our residency conference activity. In previous years, social activities such as a residency barbecue or a paintball match were participated in. This year, after several difficult site reviews and with an ongoing probation period, we wanted to do something different that promoted team-building and residency wellness. This article describes how we created a novel retreat curriculum that focused on team building and had outside activities with resident and faculty bonding. The goal was to have fun, entertaining activities that promoted bonding and that resulted in high resident satisfaction.

## Materials and methods

Our EM residency holds an annual retreat for the residents each spring. For the May 2016 retreat, the incoming chief residents asked to have a faculty-created "Amazing Race" involving events all over our city of 400,000 people. The idea of sending 25 or more type-A personalities racing around the city at breakneck speeds was frightening to program leaders. Also, much like most residency programs, our budget was tight, so we needed to find a cost-effective way to put an event like this together.

**Figure 1:**
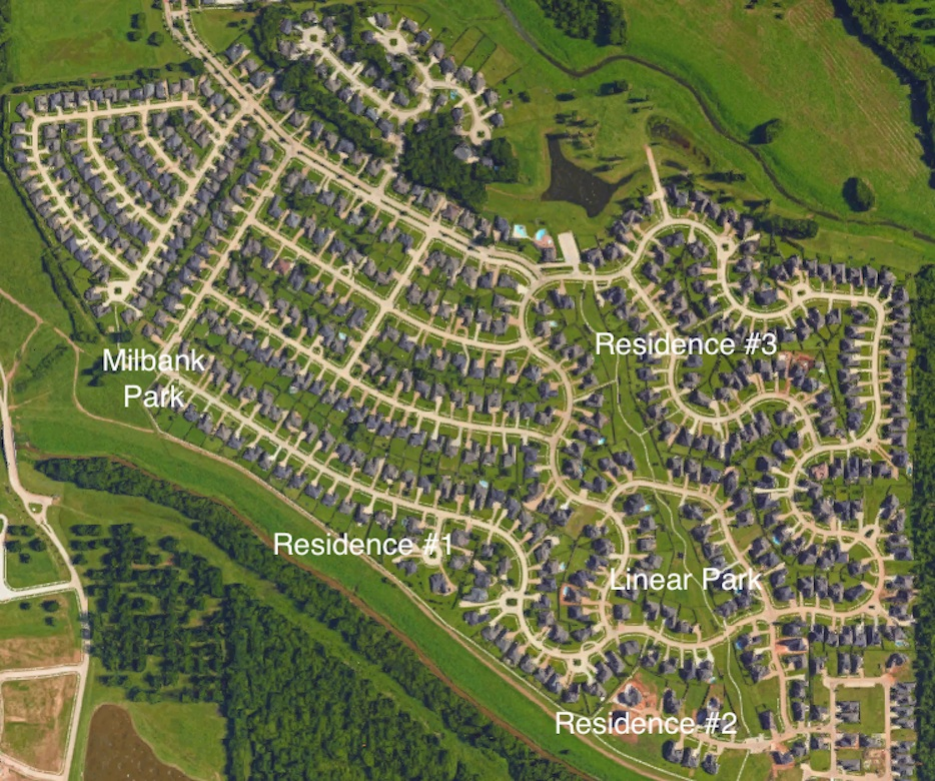
Retreat location schematic

A retreat task force was formed to create this retreat curriculum. Addressing the safety issue, we decided to hold the event in one area where participants could walk from activity to activity. After looking at many parks and venues near the medical center, we chose a local neighborhood. Three of our faculty members own homes in the neighborhood, which is situated on 300 acres and has two parks. Using this neighborhood gave us the ability to have five stations―three at faculty homes and two at the neighborhood parks (Figure 1).

The next step was to design the stations for the activity. The chief residents had requested fun team-building activities that were not necessarily related to medicine. One of activities the task force decided would be a large group game to start the morning. One of the games would address pop-culture and another faculty trivia. For the remaining two activities, a Google© search was undertaken for games that foster teamwork and trust. Two were found that matched our intent and budget.

The day of the retreat began with residents being split into four teams by having them draw lots with colored pieces of paper. The teams remained the same throughout the day. The first activity was a large group activity called “Human Hungry Hippos,” which is based on the childhood board game of “Hungry, Hungry Hippos.” In our version, each team was given a furniture dolly tied to a thin rope and a small laundry basket. In the center of the playing area were 450 plastic balls. During each round, one person for each team was the “hippo” and was rolled out by their teammates on the dolly. The “hippos” held a basket upside down and collected as many balls as possible by setting it on the ground and were pulled back to their team using the rope. This process was repeated until all the balls had been taken from the center. Then the ball counts for each team were tallied and the balls were replaced in the middle. A new “hippo” was chosen for each team and the steps were repeated. This station was scheduled for 30 minutes.

Then the teams split up, each going to one of the other four stations. Each station was 20 minutes with ten minutes walking time to the next one.

The second activity was “Watch Where You Step.” In this game, an irregularly-shaped area (greater than 6 x 12 feet) was marked off on a concrete area, upon which squeaky toys and colored pieces of paper had been placed randomly. Two members of the team put on blindfolds and then walked across the marked off area, being guided by the voices of their team members. If a blindfolded person stepped on one of the colored pieces of paper, he or she became “frozen.” Then the other blindfolded person had to be guided by voice to one of the squeaky toys to unfreeze their team member. The game continued until all team members had walked across the area.

The third activity was “Find the Common Thread.” At this station, the team members were asked to brainstorm to find one thing in common that had nothing to do with medicine. After they discovered their commonality, they had to come up with a movement or saying that would identify them for the rest of the day. For example, if all members of a group had dogs, they would bark.

For the final two activities, we wanted to do something completely original and specific to our program. We created a hybrid game of Pictionary and charades. Several faculty members wrote local and pop culture trivia questions, which the residents took turns drawing from a bag. The resident who drew the question would have to think of the answer, revealing the question and answer only to the faculty moderator. The resident then had to convey the answer in either charades or Pictionary format. Points were awarded for the number of questions answered by the resident and the number of charades/drawings that drew a correct answer from the rest of the team.

The last station was “Get to Know Your Faculty Better.” Residents were presented with trivia about the faculty members and had to match the information to the correct person. To create this station, an email message was sent to the entire faculty for interesting trivia about themselves. Eight faculty members replied with interesting personal facts, which were distilled down into 40 faculty trivia questions. Points were awarded to the team who answered the most correctly. Answers were revealed during the residents’ lunch.

The cost for the retreat was minimal. The facilities were free for our use, as they were neighborhood parks and faculty homes. Most of the expense involved the supplies for “Human Hungry Hippos,” for which we needed four furniture dollies. For our retreat, the dollies were supplied by the program director, but they could be purchased from a hardware store for approximately $20 each. One of the faculty members purchased 450 balls at a total cost of $60, which was loaned to the retreat. Supplies for all of the activities were purchased for less than $30 at a local store.

After the teams had completed all of the stations, all resident and faculty participants gathered at the home of a faculty member for the reveal of the faculty trivia answers and the awarding of prizes to the winners. A catered lunch and social time followed.

A post-retreat survey was distributed through an electronic survey (SurveyMonkey San Mateo, CA) to the residents to gauge their overall satisfaction with the retreat and with each of the activities. Review of the survey was performed by the Louisiana State University-Shreveport IRB and was determined to be not human research.

## Results

Of the 25 residents who attended, 21 answered the survey (one participant returned a survey leaving the ranking questions incomplete). The responses were arranged on a Likert scale of one through five, with five indicating “strongly agree” and one indicating “strongly disagree.” The residents considered the team-building activities a fun way to generate camaraderie, giving it an average rating of 4.10. They indicated that the team-building activities made them feel they were contributing to the achievement of a group goal or objective, giving it an average rating of 4.15. The average score for the overall retreat experience was 4.10.

## Discussion

In 2013, Hills stated that one of the best ways to improve the performance of a medical practice team is to hold a team retreat [7]. One of the major goals of a retreat is to encourage socialization in an informal setting, allowing barriers to be broken down and improving teamwork [8]. Teamwork is an important skill, especially in emergency medicine, where colleagues depend on each other to keep the department running when one person becomes involved in a critical situation.

Burnout is a state of exhaustion of emotional or physical strength or even motivation, usually resulting from prolonged stress or frustration. In residency, stress and frustration are significant. The combination of exhaustion and stress can lead to increased rates of physician error. In 2005, Prins, et al. showed increased rates of error in residents reporting moderate to severe burnout. Those with severe burnout reported more errors [8]. Burnout can also lead to sub-optimal patient care [9]. In 2014, the ACGME recognized the magnitude of this problem and convened a symposium to address resident wellness. Recommendations to make the residency practice environment better included more faculty-trainee interaction, designated and protected social time and team building, a competitive environment that supports learning from one another, promotion of exercise and life/work balance [10].

Residency burnout has become an important topic in recent years. Ghetti, et al. found that more than 55% of obstetrics/gynecology interns in their study group reported burnout symptoms [11]. Other investigators found moderate to severe burnout in >70% of residents [12-13]. When comparing specialties, one of the highest levels of burnout in practicing physicians is in the field of emergency medicine, which induces particular concern because the burnout probably started in residency [14]. So how can we prevent and not just alleviate burnout? Retreats provide one preventative avenue because they build strong relationships between the residents and lower the likelihood of burnout. Stronger bonds enable residents to recognize signs of burnout and depression in their colleagues.

The pressures that lead to burnout can also lead to cynicism, loss of humanitarian ideals, and even depression [15]. In the 1970s and 1980s, the problem of stress in residency leading to burnout, fatigue, and depression in residents was recognized and programs were developed to detect, control, and mitigate it. Despite these efforts, in 2002 Collier, et al. showed that more than one-third of residents reported four or five depressive symptoms [16]. In 2009, a study of more than 2000 medical students and residents detected probable mild/moderate depression in 9.2% of them and probable major depression in 12% [17]. This level of depression has led to 300 to 400 physician suicides each year [18]. Combatting burnout is not enough: we need to address overall resident wellness.

In 2003 and again in 2011, the ACGME enacted duty hour restrictions to combat burnout and fatigue. However, in 2013 it was shown that >50% of residents surveyed were fatigued and >75% had criteria for burnout [19]. Clearly, duty hour restrictions have not been the cure for this problem, so the conversation needs to turn to new and different activities to tackle this problem such as this novel retreat activity.

There are several limitations for this study. First, the resident’s reactions were gathered by survey which could lead to reporting bias. Second, the study favorably reported the resident’s satisfaction with the team building and comradery and this was used as an indicator of the impact this retreat had on their wellness. Third, this was a limited group at one residency program so it may not be generalizable to other residencies or groups. 

## Conclusions

With the spotlight being placed on resident wellness, we as residency leaders need to combat the issues of stress, fatigue, and burnout in new and imaginative ways. A simple way to begin to address wellness in your department is a resident retreat. We developed a retreat that incorporated team-building, resident bonding, and faculty-resident bonding. It is possible to design a novel retreat with minimal financial expenditure that will yield high satisfaction among residents and increased interaction with faculty members.
